# Impact of maternal lateral tilt on cardiac output during caesarean section under spinal anaesthesia: a prospective observational study

**DOI:** 10.1186/s12871-022-01640-6

**Published:** 2022-04-11

**Authors:** Chiara Sonnino, Luciano Frassanito, Alessandra Piersanti, Pietro Paolo Giuri, Bruno Antonio Zanfini, Stefano Catarci, Gaetano Draisci

**Affiliations:** Unit of Obstetric and Gynecologic Anesthesia, IRCCS Fondazione Policlinico Universitario Agostino Gemelli, Rome, Italy

**Keywords:** Left uterine displacement, Cardiac output, Noninvasive hemodynamic monitoring, Cesarean delivery, Spinal anesthesia

## Abstract

**Background:**

Left uterine displacement (LUD) has been questioned as an effective strategy to prevent aortocaval compression after spinal anesthesia (SA) for cesarean delivery (CD). We tested if LUD has a significant impact on cardiac output (CO) in patients undergoing CD under SA during continuous non-invasive hemodynamic monitoring with Clearsight.

**Methods:**

Forty-six patients were included in the final analysis. We considered 4 timepoints of 5 min each: T1 = baseline with LUD; T2 = baseline without LUD; T3 = after SA with LUD; T4 = after SA without LUD. LUD was then repositioned for CD. The primary outcome was to assess if CO decreased from T3 to T4 of at least 1.0 L/min. We also compared CO between T1 and T2 and other hemodynamic variables: mean, systolic and diastolic blood pressure (respectively MAP, SAP and DAP), heart rate (HR), stroke volume (SV), stroke volume variation (SVV), pulse pressure variation (PPV), contractility (dP/dt), dynamic arterial elastance (Ea_dyn_) at the different timepoints. Data on fetal Apgar scores and umbilical arterial and venous pH were collected.

**Results:**

CO did not vary from T3 to T4 (CO mean difference -0.02 L/min [95% CI -0.88 to 0.82; *P* = 1). No significant variation was registered for any variable at any timepoint.

**Conclusions:**

LUD did not show a significant impact on CO during continuous hemodynamic monitoring after SA for CD.

**Trial registration:**

(retrospectively registered on 03/12/2021) NCT05143684.

## Introduction

Since 1953, the gravid uterus in pregnancies at term has been recognized as a cause of aortic and caval compression in the supine position [1, 2]. Later, experiments with venograms provided a visual evidence of the impaired venous return suggesting the adoption of the left uterine tilt in clinical practice [3]. In most patients, venoconstriction of the lower limbs allows complete compensation [4], but sympathetic blockade following spinal anesthesia (SA) for cesarean delivery (CD) blunts the cardiovascular compensatory mechanisms, exacerbating maternal hypotension and neonatal depression [5–7].

The introduction of a 15° left uterine displacement (LUD) was proposed for the first time by Crawford and colleagues in 1972, as a result of their experiments on 150 women undergoing CD under general anesthesia [8]. However, there is no consensus on whether tilting the table improves maternal or neonatal outcome. In fact, not only LUD is rarely effectively achieved in every day practice [7, 8], making its efficacy in preventing aortocaval compression unreliable, but it may make the operation more difficult for the surgeon.

The introduction of an optimized vasopressor and fluid therapy posed questions on its effective utility [9–11].

A Cochrane review found no differences in hypotensive events between supine and LUD patients [12].

Lee and colleagues measured CO, stroke volume (SV) and systemic vascular resistances by suprasternal Doppler ultrasound in not anesthetized parturients with four levels of left lateral tilt (0°, 7.5°, 15° and 90°) [13], showing that aortocaval compression can be effectively minimized by the use of a left lateral tilt of 15° or greater.

On the other hand, Tsai and colleagues showed that NICOM hemodynamic monitoring could not detect any difference in cardiac index between patients with LUD and supine patients [14]; while Chungsamarnyart showed only modest hemodynamic advantages (higher CO, less hypotension, higher dP/ dT) with pre-delivery LUD [15].

The aim of this prospectic observational study was to evaluate if CO decreased of at least 1.0 L/min after removing LUD after SA for CD during continuous non-invasive monitoring. We also compared values of mean arterial pressure (MAP), systolic arterial pressure (SAP), diastolic arterial pressure (DAP), SV, stroke volume variation (SVV), heart rate (HR), pulse pressure variation (PPV), contractility (dP/dt_max_) and dynamic arterial elastance (Ea_dy*N* =_ PPV/SVV) with and without LUD before and after SA to asses for significative differences.

## Matherials and Methods

This trial was conducted from 1 June 2020 to 31 July 2020 at the delivery suite of Agostino Gemelli University Hospital IRCCS of Rome, Italy, in accordance with Good Clinical Practice guidelines, the principles of the Declaration of Helsinki, and relevant regulatory requirements. The trial was retrospectively registered in ClinicalTrial.gov, identifier NCT05143684 on 03 December 2021 and it was approved by the Internal Ethic Committee (ID 3197, protocol N 27861/2020).

Written informed consent was obtained from each participant.

We included adult (≥ 18 years old) pregnant patients at term (36th to 40th week of gestation) scheduled for elective CD under SA, who, in addition to standard monitoring (5-lead electrocardiogram, pulse oximetry, non-invasive intermittent blood pressure, urine output), underwent perioperative non-invasive monitoring by ClearSight system on the Edwards Lifesciences HemoSphere platform (Edwards Lifesciences, Irvine, CA).

Exclusion criteria were: American Society of Anesthesiologists status > 3, cardiac arrhythmias or aortic regurgitation, pregnancy-induced hypertension, pre-eclampsia, body mass index (BMI) > 35 kg/m^2^, fetal complications, coagulation disorders or contraindication to neuraxial block, emergency surgery, preoperative infection, patient’s refusal.

The ClearSight system consists of a finger cuff positioned at the middle phalanx of the third finger of the non-dominant hand of the patient, able to detect continuous noninvasive blood pressure and advanced hemodynamic parameters [16].

The parameters we evaluated from the ClearSight system for the analysis were CO, MAP, SAP, DAP, SV, SVV, HR, PPV, dP/dt_max_ and PPV/SVV recorded at 20 s-intervals.

All patients had a peripheral vein cannulated in the pre-anesthesia room and received metoclopramide 10 mg, pantoprazole 40 mg and cefazoline 2 gr delivered with a total of 100 ml of normal saline. Fluids were then stopped until spinal anesthesia. Anesthesia was delivered in sitting position using a 25-G Whitacre spinal needle, at the L3-4 vertebral interspace, with hyperbaric 0.5% bupivacaine, sufentanil 5 mcg and morphine 100 mcg. The bupivacaine dose administered was standardized according to patient’s height, as usual practice in our Institution: 8 mg for women < 160 cm tall, 9 mg for women between 160 and 170 cm, and 10 mg for those > 170 cm. Once the anesthetic procedure was completed, all patients received a rapid crystalloid co-load of 7 ml/kg over 10 min. During surgery and after delivery, fluid management was left to the attending anesthesiologist.

**Fig. 1:**
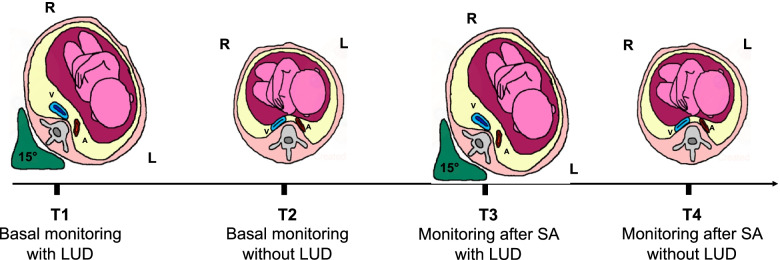
Description of timepoints for comparison of hemodynamic variables. V: vena cava; A: aorta; LUD: left uterine displacement; SA: spinal anesthesia. R = right; L = left

We considered 4 timepoints. We indicated as T1 the baseline values recorded for 5 min, after initial stabilization of parameters, with the patient laying down on the operating table with LUD. At T2, LUD was removed and we considered for the analysis hemodynamic data of the subsequent 5 min. We indicated as T3 the 5 min following SA with a satisfactory sensory block and as T4 the subsequent 5 min following LUD removal. Figure 1 summarizes the timepoints of our analysis.

LUD was accomplished by positioning a wooden wedge and wrapped with cotton, to make it comfortable, and medical sheets with a measured angle of 15° under the right flank of the laying down patient. The correct lateral tilt inclination was measured with a bubble level. In all patients, after T4, the 15° wooden wedge was repositioned in all patients and surgery was performed with LUD.

The attending anesthesiologist was blinded to the advanced hemodynamic parameters from the ClearSight system except for the continuous BP values. We defined hypotension as an absolute value of MAP < 65 mmHg. This value was considered as trigger for the attending anesthesiologist for the administration of norepinephrine 5 mcg. Norepinephrine boluses were repeated to reach a MAP > 65 mmHg. Bradycardia was defined as a heart rate of < 60 bpm. Atropine 0.5 mg was administered for the treatment of bradycardia combined with hypotension, or for an absolute value of heart rate < 45 bpm for more than 20 s. After delivery, Oxytocin was administered to facilitate the uterine contraction.

We also evaluated the impact of maternal blood pressure and CO on fetal outcome collecting neonatal Apgar scores at 1 and 5 min after birth, and umbilical cord arterial and venous pH.

## Statistical Analysis

We estimated the sample size based on the CO reported in a recently published randomized controlled trial comparing patients with and without LUD during CD under SA [15]. The reported mean CO was 7.20 ± 1.78 L/min in patients with LUD and 6.23 ± 1.44 L/min in patients without LUD.

Considering a significance level of 0.05 and a power of the test of 0.90 (https://clincalc.com/stats/samplesize.aspx), we estimated a minimum sample size of 35 patients to detect the same variation of CO after LUD removal. We included all patients whose data were recorded and complete, who did not meet any exclusion criteria, for a total of 46 patients, to account for dropouts.

Continuous variables were reported as mean (± standard deviation, SD) if normally distributed, and as median and interquartile range (IQR) or as median (minimum–maximum) if not normally distributed. Categorical variables were reported as number and percentage. Shapiro–Wilk test was used to assess normality of data distribution and the equality of variances was verified with the variance ratio test. Repeated measure analysis of variance (ANOVA) or its non-parametric alternative Friedman test for non-normally distributed variables were used to compare patients’ longitudinal hemodynamic data from baseline until ten minutes after SA was performed. Sphericity assumption was not violated and it was assessed with the Mauchly’s test (*p* > 0.05). Bonferroni’s adjustments for multiple comparisons was applied for pairwise comparisons among group means.

The statistical significance level was set at 0.05%.

## Results

**Fig. 2:**
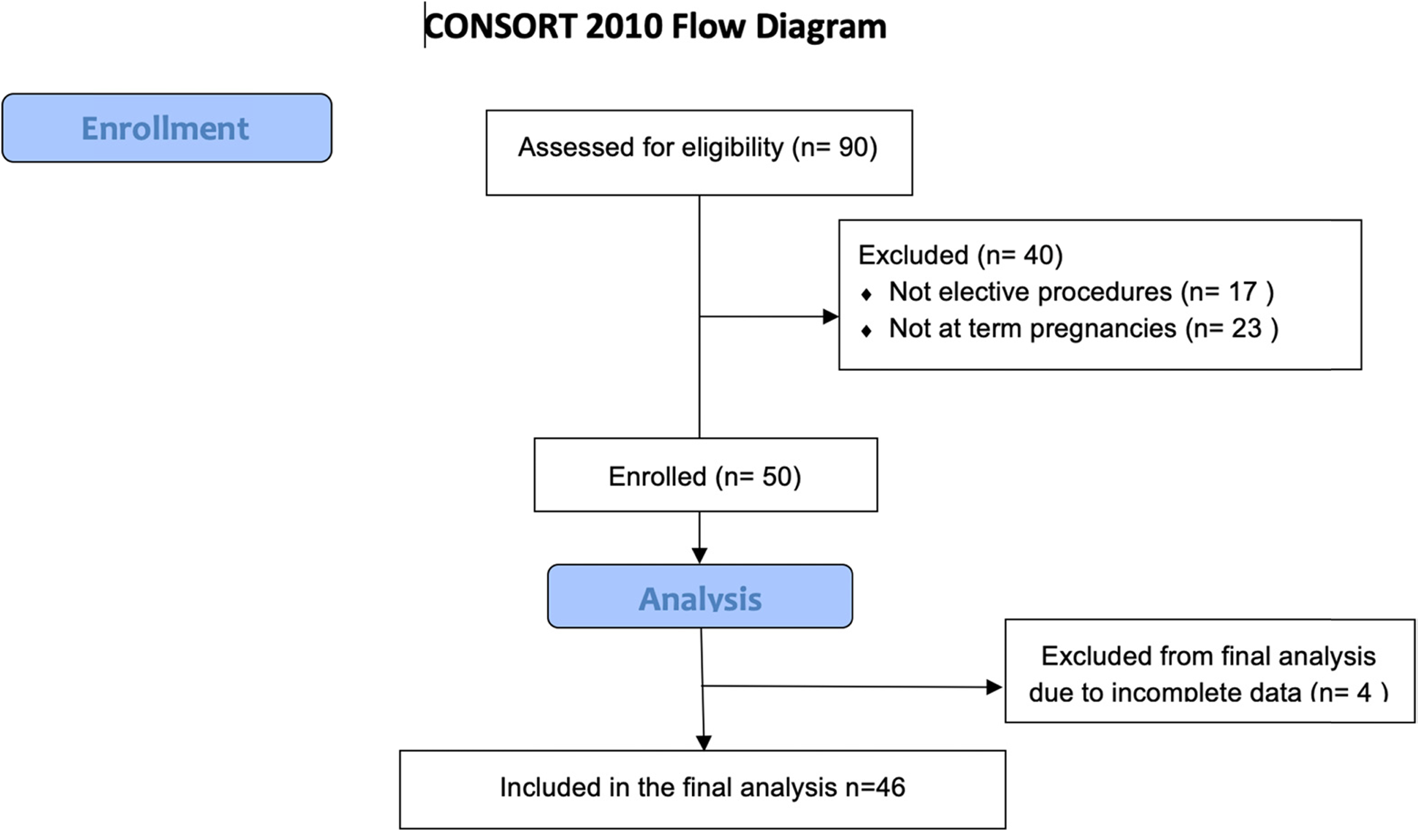
Consort diagram

We screened 90 pregnant women at term scheduled for elective CD. Forty cases were excluded because they did not meet the inclusion criteria (not at term *N* = 23, not elective procedures *N* = 17). Four patients were excluded due to incomplete data records. We used for the final analysis data from 46 women, including 6 twin pregnancies with mean gestational age of 38 ± 2 weeks. The consort diagram is represented in Fig. 2. All the included patients had successful sensory block at T4 that allowed surgery to be completed.

Demographic and intraoperative data, together with fetal Apgar scores and umbilical venous (UV) and arterial (UA) blood gas analysis are summarized in Table 1.

**Table 1:** - Demographic and intraoperative data of all patients. Data are expressed as n (%), mean ± SD or median (range). LUD: left uterine displacement

	***N* = 46**
Age (year)	36 (± 6)
Height (m)	1.63 (± 0.1)
Weight (Kg)	74 (± 12)
Body Mass Index (Kg/m2)	27 (24–30)
Twin pregnancy	6 (13%)
0.5% hyperbaric bupivacaine dose (mg)	9 (± 1)
Crystalloid co-load after neuraxial anesthesia (mL)	516 (± 83)
Norepinephrine dose after neuraxial anesthesia with LUD (mcg)	13 (± 6) ( *N* = 22)
Norepinephrine dose after neuraxial anesthesia without LUD	9 (± 6) (*N* = 20)
Total norepinephrine dose during surgery (mcg)	35 (15–50) (*N* = 38)
Operative time (min)	84 (± 16)
Apgar 1 min	8.6 (2–9)
Apgar 5 min	9.4 (2–10)
UVpH	7.32 (± 0.05)
UApH	7.29 (± 0.09)

**Fig. 3:**
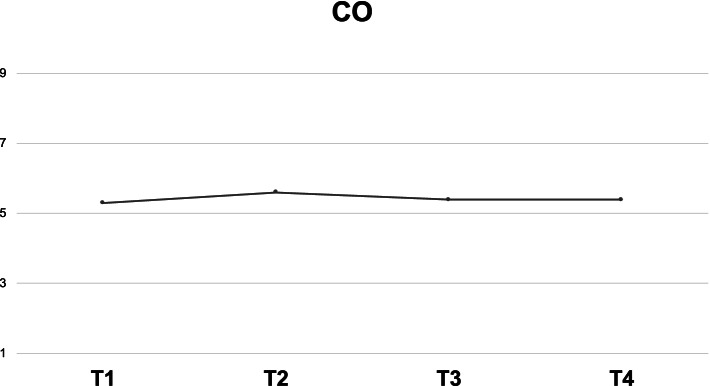
Mean values of CO at the single timepoints in ml/min. CO = cardiac output. T1 = baseline with left uterine displacement (LUD); T2 = baseline without LUD; T3 = after spinal anesthesia (SA) with LUD; T4 = after SA without LUD

We did not find any significant CO variation after LUD removal after SA, nor after LUD removal at baseline (CO mean difference 0.34 (SE 0.32) L/min [95% CI -0.05; 1.19] at baseline and -0.028 (SE 0.32) L/min [95% CI -0.08; 0.82] after SA (*P* = 1.0) (Fig. 3).

**Fig. 4:**
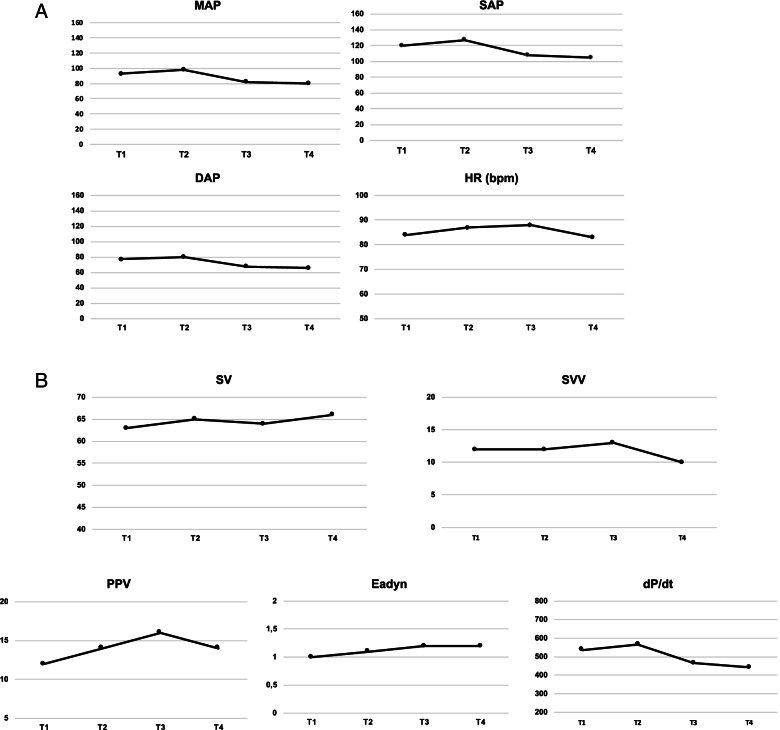
Mean values of main hemodynamic variables at the single timepoints: T1 = baseline with left uterine displacement (LUD); T2 = baseline without LUD; T3 = after spinal anesthesia (SA) with LUD; T4 = after SA without LUD. SA*P* = systolic arterial pressure; MA*P* = mean arterial pressure; DA*P* = diastolic arterial pressure; HR = heart rate ; SV = stroke volume; SVV = stroke volume variation; PPV: pulse pressure variations; dP/dt: contractility in mmHg/sec; Ea_dyn =_ dynamic elastance

We did not find a reduction in CO after LUD removal after SA of at least 1.0 L/min (CO mean difference -0.02 L/min [95% CI -0.88 to 0.82]), nor after LUD removal at baseline (CO mean difference 0.34 (SE 0.32) L/min [95% CI -0.05; 1.19]) and -0.028 (SE 0.32) L/min [95% CI -0.08; 0.82] after SA (*P* = 1.0) (Fig. 3). There was no significant variation of the other variables analysed at any timepoint (Fig. 4).

All the hemodynamic values at different timepoints are reported in Table 2.

**Table 2:** – Hemodynamic variables at the different timepoints. Data are expressed as mean ± SD or median (range). Cardiac Contractility assessed as dP/dtmax. Dynamic Arterial Elastance (Eadyn) assessed as PPV/SVV. LUD: left uterine displacement; SA: spinal anesthesia; UVpH: umbilical vein pH; UApH: umbilical artery pH. T1 = baseline with LUD; T2 = baseline without LUD; T3 = after SA with LUD; T4 = after SA without LUD

	**T1**	**T2**	**T3**	**T4**
CO (L/min)	5.3 (± 1.5)	5.6 (± 1.4)	5.4 (4.3–6.4)	5.4 (4.2–6.5)
MAP (mmHg)	93 (± 8)	98 (± 8)	82 (75–89)	80 (71–86)
SAP (mmHg)	120 (± 13)	127 (± 12)	108 (99–118)	105 (95–113)
DAP (mmHg)	77 (± 7)	80 (± 7)	68 (64–74)	66 (60–71)
HR (bpm)	84 (± 11)	87 (± 11)	88 (± 14)	83 (± 14)
SV (ml/b)	63 (± 16)	65 (± 15)	64 (52–72)	66 (52 -76)
SVV (%)	12 (10–14)	12 (11–14.6)	13 (11–14)	10 (9–12)
PPV (%)	12 (9–15)	14 (13–17)	16 (± 4)	14 (± 4)
dP/dt (mmHg/sec)	536 (426–621)	566 (501–671)	466 (383–603)	443 (36–528)
Eadyn	1 (0.9–1.2)	1.1 (1.0–1.2)	12 (1.1–1.4)	1.2 (1–1.5)

At T3, during the first 5 min after SA, 22 patients received norepinephrine to treat hypotension and at T4, during the subsequent 5 min after removal of LUD, vasopressor was administered to 20 patients [mean dose 13 (± 5.87 SD) mcg at T3 vs. 9 (± 5.91 SD) mcg at T4; *P* = 0.06]. Of these patients, 7 received norepinephrine at T3 and at T4. 14 patients (30%) did not need vasopressors during the first 10 min after SA. No patient experienced nausea or vomiting. Only one patient had bradycardia which required atropine. There were no episodes of cardiac arrest.

Mean Apgar scores at 1 and 5 min were, respectively, 8.6 (min 2; max 9) and 9.4 (min 2; max 10), with one case of Apgar score of 2 at 1 and 5 min in a baby with trisomy 18 disease born at 36 weeks of gestation, which was undiagnosed until birth in a patient with poor assistance during pregnancy. Mean UV pH was 7.32 (± 0.05), and mean UA pH was 7.29 (± 0.09).

## Discussion

In this prospective observational study, we found that under continuous hemodynamic monitoring, CO did not show any significant variation after LUD removal under SA for CD. LUD showed no impact on CO neither at baseline, before SA. Blood pressure, HR, SV, SVV, PPV, dP/dt_max_ and Ea_dyn_ did not vary significantly with and without LUD either at baseline or after SA.

Of 46 patients, 22 (48%) needed vasopressor support right after SA with LUD, and 20 (43%) needed vasopressor support after LUD removal under SA. The total amount of norepinephrine was significantly higher after LUD removal under SA, but SAP, MAP and DAP were not significantly influenced. This may suggest that, even if LUD may have a role in maintaining MAP, prompt vasopressor administration is able to correct hypotensive events even without LUD. The continuous blood pressure monitoring and the prompt medical intervention triggered by a conservative threshold (MAP < 65 mmHg) allowed an efficient hemodynamic control, as demonstrated by the lack of emetic symptoms, such as nausea and vomiting. We should consider that in everyday practice blood pressure during CD is not measured continuously, but international recommendations suggest non-invasive blood pressure measurements every minute and prophylactic vasopressor infusion [17]. In 2017, Lee and co-authors showed that optimal fluid and vasopressor therapy controlled the component of hypotension due to the aortocaval compression by the gravid uterus without consequences for the foetus [11].

Previous studies showed mixed results on the hemodynamic impact of LUD [8–20]. A Cochrane review showed that LUD did not have any impact on non-invasively measured blood pressure [12]. Some authors with hemodynamic monitoring showed that LUD determined minimal improvement in term pregnancies without anesthesia [14, 15], while Lee and colleagues reported a better hemodynamic profile with LUD [11]. In women undergoing SA for CD, LUD showed to improve CO and the overall hemodynamic equilibrium [15].

Most studies on maternal hemodynamics focused on systolic blood pressure. This parameter is reliable and easily reproducible, hence its wide use. CO is not routinely measured in elective cesarean deliveries, and non-invasive monitoring devices are expensive and not widely available. On the other hand, CO is a better indicator of fetal perfusion than blood pressure, due to the changes in peripheral resistances that occur in pregnancy, which do not necessarily reflect fetal perfusion [21].

Recently, Chungsamarnyart and colleagues published their randomized-controlled trial comparing non-invasive monitoring of CO in patients with LUD and without LUD, showing that LUD provided modest hemodynamic advantages (higher CO, less hypotension, higher dP/dT) pre-delivery. The results support maternal hemodynamic benefits of LUD until delivery in women with term pregnancies undergoing CD with SA [15].

Preventing hypotension and hemodynamic derangement after SA for CD is a challenge for the obstetric anesthesiologist in order to avoid maternal and fetal complications.

In this study we open new questions on the hemodynamic benefit of LUD, suggesting that preventive vasopressor therapy and optimized fluid management may allow an optimal uterine perfusion (as shown by the maintenance of CO values after LUD removal) independently from aortocaval compression.

This study has some limitations. Firstly, its design does not include a control group, but patients act as their own control after LUD removal before and after SA. On the other hand, the continuous hemodynamic monitoring allowed to better evaluate the impact of LUD on CO with standard anesthetic management, correcting for inter-individual variables.

Also, we defined hypotensive events as MAP < 65 mmHg, even if in obstetric anesthesia the most common definition of hypotension refers to SAP (< 80% baseline or < 100 mmHg) [22, 23]. Nevertheless, the role of MAP as determinant of organ perfusion is well known [23, 24].

## Conclusions

CO did not decrease significantly after LUD removal in patients under SA for CD during continuous hemodynamic monitoring. Optimization of fluid and vasopressor therapy may be sufficient to prevent aorto-caval compression by the gravid uterus and the consequent reduction of venous return after SA for CD.

## Data Availability

The datasets used and/or analysed during the current study are available from the corresponding author on reasonable request.

## Abbreviations

LUD: Left uterine displacement
SA: Spinal anesthesia
CD: Cesarean delivery
CO: Cardiac output
MAP: Mean arterial pressure
SAP: Systolic arterial pressure
DAP: Diastolic arterial pressure
HR: Heart rate
SV: Stroke volume
SVV: Stroke volume variation
PPV: Pulse pressure variation
dP/dt: Contractility
Ea_dyn_: Dynamic arterial elastance
SD: Standard deviation
IQR: Interquartile range
UV: Umbilical venous
UA: Umbilical arterial
